# *Euglena* Central Metabolic Pathways and Their Subcellular Locations

**DOI:** 10.3390/metabo9060115

**Published:** 2019-06-14

**Authors:** Sahutchai Inwongwan, Nicholas J. Kruger, R. George Ratcliffe, Ellis C. O’Neill

**Affiliations:** Department of Plant Sciences, University of Oxford, South Parks Road, Oxford OX1 3RB, UK; sahutchai.inwongwan@new.ox.ac.uk (S.I.); nick.kruger@plants.ox.ac.uk (N.J.K.); george.ratcliffe@plants.ox.ac.uk (R.G.R.)

**Keywords:** *Euglena*, central metabolic pathway, subcellular location

## Abstract

Euglenids are a group of algae of great interest for biotechnology, with a large and complex metabolic capability. To study the metabolic network, it is necessary to know where the component enzymes are in the cell, but despite a long history of research into *Euglena*, the subcellular locations of many major pathways are only poorly defined. *Euglena* is phylogenetically distant from other commonly studied algae, they have secondary plastids bounded by three membranes, and they can survive after destruction of their plastids. These unusual features make it difficult to assume that the subcellular organization of the metabolic network will be equivalent to that of other photosynthetic organisms. We analysed bioinformatic, biochemical, and proteomic information from a variety of sources to assess the subcellular location of the enzymes of the central metabolic pathways, and we use these assignments to propose a model of the metabolic network of *Euglena*. Other than photosynthesis, all major pathways present in the chloroplast are also present elsewhere in the cell. Our model demonstrates how *Euglena* can synthesise all the metabolites required for growth from simple carbon inputs, and can survive in the absence of chloroplasts.

## 1. Introduction

Euglenids, a group of unicellular flagellate algae, have long been studied for their biochemistry, physiology, anatomy, and industrial potential, due to the remarkable metabolic plasticity that allows them to grow in a wide range of conditions [1]. *Euglena* can harness energy heterotrophically, mixotrophically, and photo-autotrophically, and its cultivation is relatively easy, fast, and well established. Euglenids can be found in a broad range of ecological niches including fresh water, brackish water, snow, high and low pH conditions, and both aerobic and anaerobic environments [2]. *Euglena gracilis* is the most studied species of *Euglena* and is regarded as a useful model organism for studying cell biology and biochemistry. Euglenids were once considered one of the most ambiguous groups in terms of evolution and metabolic operation, due to the combination of both “plant-” and “animal-” like features [3]. They are now classified into the kingdom Excavata, superphylum Discoba, subphylum Euglenozoa. *Euglena* is one of the very few plastid-containing organisms for which complete loss of the chloroplast is not lethal. Even the human parasitic apicomplexans retain their plastids for the synthesis of isoprenoids, fatty acids, and heme, while in non-photosynthetic, parasitic plants plastids are necessary for aromatic amino acid biosynthesis and are involved in starch synthesis [4]. Whilst these plastid-localised pathways can be targeted to kill such organisms, *Euglena* can survive complete loss of the plastid and the biochemical explanation for this remains to be established.

The genome of *E. gracilis* is estimated to be around 500 Mb in size, with large amounts of highly repetitive sequences [5], which leads to difficulty in genome sequencing and analysis. The structural complexity of the genome has arisen from a series of horizontal gene transfers and endosymbiosis events throughout its evolutionary history, causing difficulty in classifying euglenids using modern molecular techniques [6]. A study of the distribution of the homologues of 2770 expressed sequence tags (ESTs) from *E. gracilis* has shown that euglenids are closely related to the kinetoplastids [7]. Euglenids first split from the ancestral Euglenozoa, a eukaryotic protozoa, around a billion years ago [8]. After the endosymbiotic transfer of genes from a hypothesized, since-lost, red algal endosymbiont to the nuclear genome [9], a eukaryotic green alga endosymbiont was incorporated [10], bringing many genes involved in the function and maintenance of the chloroplast. The transcriptome of *Euglena* suggests that many other genes were acquired from diverse distantly related species and the genetic control mechanisms in *Euglena* involve genes which are as sophisticated as those in animal and plant eukaryotes [11].

*Euglena* is considered to be a promising organism for industrial application due to its ability to produce various nutrients and bioactive compounds, such as proteins, polyunsaturated fatty acids, vitamin A, vitamin C, and β-1,3-glucan [12]. The application of *Euglena* in environmental engineering has been studied for wastewater treatment systems, energy sources and bioindicators for environmental pollutants. *Euglena* sp. isolated from sewage treatment plants had higher nutrient removal capability and growth rate than other algae [13]. These results indicate that *Euglena* could be considered as a viable source for biofuel production from wastewaters.

There is no doubt that *E. gracilis* is an interesting organism in terms of its evolution, metabolic capacity, and application and has thus been the subject of intense study. Due to its extraordinary metabolic capacity, investigating and understanding the *Euglena* metabolic network could help expand the applications of this organism and shed light on several mysteries of evolution and secondary endosymbiosis. Investigation of the metabolism of *Euglena* requires the definition of the metabolic network, whether at genome scale for flux balance analysis, or at the level of core metabolism for metabolic flux analysis. This would allow the metabolic phenotype of the organism to be investigated in much the same way as in highly compartmented plant cells [14]. In organisms with complex evolution like *Euglena*, even though the central metabolic pathways are conserved, the characteristics and subcellular localisation of the enzymes involved in the pathway can differ. This is particularly true for *Euglena*, where the secondary chloroplast has a relatively recent evolutionary origin (~600 MYA [15]) and a unique third plastid membrane, giving rise to a novel subcellular compartment in this intermembrane space.

Here, we provide an overview of the central metabolic pathways in *Euglena gracilis*, highlighting unique features. We assess the reported subcellular location of enzyme activities and proteins in *Euglena* and propose a model of the organisation of the central metabolic network.

## 2. Results

### 2.1. Pathway Localisation from Sequence Information

Even though *Euglena* has long been studied for its biotechnological potential, its genetic and metabolic capacities are poorly established due to the size and complexity of its genome. In the absence of an annotated genome sequence for any species of *Euglena*, transcriptome sequencing has been used as the preliminary alternative to genome structure analysis, with the aim of providing data on gene expression and regulation under different conditions [16,17].

#### 2.1.1. Metabolic Pathways in *Euglena*

The earliest reported extensive transcriptomic analysis of *E. gracilis* studied cells grown in dark and light conditions and illustrated the versatile metabolic capacity of *Euglena* [16]. All the core pathways of carbohydrate metabolism and photosynthesis were identified, including glycolysis, gluconeogenesis, the tricarboxylic acid cycle (TCA), the pentose phosphate pathway (PPP), and the Calvin cycle. In addition, the pathways for production of other major classes of compounds including carotenoids, thylakoid glycolipids, fatty acids, and isoprenoids were also identified in the transcriptome. Besides the evidence for lipid, amino acid, carbohydrate, and vitamin metabolism, the transcriptome also revealed the capacity of *E. gracilis* to produce multifunctional polydomain proteins that relate to those from both fungi and bacteria and may have been obtained by horizontal gene transfer during its evolution [11]. Furthermore, the transcriptome showed the capacity for polyketide and non-ribosomal peptide biosynthesis [18], along with capacities for using the pathways for vitamin C, vitamin E, and glutathione metabolism to respond to stresses. A subsequent comparative study of the transcriptome of *E. gracilis* under aerobic and anaerobic conditions investigated the regulatory system of wax ester metabolism [17]. The metabolic network of *Euglena mutabilis* has been reconstructed using assembled transcript sequences and topology gap filling [19]. The initial draft network was incomplete with many missing reactions and could not simulate the heterotrophic growth of *E. mutabilis* in the dark [19], despite the long documented capacity of this species to do so. In combination, these studies demonstrate that the genomes of *Euglena* have features in common with genomes from both phototrophic and heterotrophic organisms, and these features provide *Euglena* with the metabolic capacity to adapt to a wide variety of conditions. These studies also demonstrate that transcript abundance does not vary greatly under different growth conditions and does not correlate with protein abundance. Thus, exploration of the metabolic capacity of *Euglena* using an exclusively transcriptomic approach is unlikely to be sufficient to understand pathway control.

#### 2.1.2. Metabolic Pathways in the *Euglena* Plastid

The chloroplast genome of *E. gracilis* has been sequenced [20] and is very similar to that of higher plants in its gene content, although the structure and evolution is different [21]. As with other organisms, the acquisition of the plastid came with many gene loses and gene transfers from the endosymbiont to the host genome [22]. The expression level of plastid genes was found to respond to environmental stimuli [23] and the rate of protein synthesis by the *E. gracilis* plastid in the dark is extremely low compared to that in the light [5,24].

As in the primary plastids of other organisms, most of the *Euglena* secondary plastid proteome is encoded in the nuclear genome. However, since the plastid of *Euglena* was acquired through secondary endosymbiosis of a photosynthetic eukaryote, its chloroplasts are surrounded by three membranes [25,26]. Thus, hundreds of plastidic proteins synthesized in the cytosol have to be transported through either three or four membranes to reach their destination in the plastid stroma or the thylakoid lumen [27] and we have no knowledge of the metabolic capabilities of the unique intermembrane space, found in no other group of organisms.

#### 2.1.3. Predicting the Subcellular Location of *Euglena* Proteins

Most of the previously published studies of the subcellular compartmentation of *Euglena* enzymes have relied on subcellular fractionation of organelles and measurement of enzyme activity distributions. Very few studies have exploited complementary molecular techniques to investigate localisation in *Euglena*. In principle, eukaryotic protein subcellular location prediction tools could be useful. To test this, the protein sequences of selected marker enzymes with defined compartmentation were analysed using a subcellular location prediction work flow. These included proteins known to be located in the chloroplast, mitochondria, cytosol, or directed through the secretory pathway. The predicted amino acid sequences of these marker proteins were deduced from the *E. gracilis* transcriptome [16]. In total 28% of these sequences had spliced leader sequences, indicated in bold in Table 1, Table 2 and Table 3. Two programs were used to predict the subcellular localisation of all the matching *E. gracilis* protein sequences, WoLF PSORT [28], and TargetP 1.1 [29]. Due to the potential presence of plant and non-plant targeting signals on *Euglena* proteins (arising from the complex evolutionary origin of *Euglena* genes), these analyses were conducted using plant, animal, and fungal reference databases in WoLF PSORT and both plant-based and nonplant-based prediction modes in TargetP 1.1. Moreover, since transport of proteins into *Euglena* chloroplasts requires transit via the secretory pathway [27,30,31], any sequence that was predicted to contain a secretion signal based on the plant-based algorithm in TargetP 1.1 was subjected to extended analysis in which the signal sequence was removed and the prediction process repeated to establish the ultimate predicted location of the mature protein.

##### Mitochondrial Targeting

The mitochondrial marker enzymes are all well-established biochemical markers and are only detected in mitochondrial fractions in *Euglena*, with the exception of isocitrate dehydrogenase which is also detected in the cytosol. At least one isoform of each of these enzymes is predicted to be targeted to mitochondria using TargetP and WoLF PSORT in all modes (see Table 1). However, using the plant-based algorithm in WoLF PSORT there was more support for some of these enzymes being in the chloroplast. One isoform of succinic semialdehyde dehydrogenase, containing a spliced leader sequence, appears to have no targeting signal and so would be predicted to be in the cytosol. One isoform of isocitrate dehydrogenase has no predicted targeting, in line with biochemical evidence for some cytosolic activity of this enzyme.

##### Proteins without Targeting Signals

Cytosolic marker proteins were selected that are routinely used as marker enzymes in subcellular fractionation studies. Overall, these had less confident predictions and some weak predictions for mitochondrial targeting (Table 2). The exception is thiosulfate sulfurtransferases, for which three isoforms had plastid targeting sequences in WoLF PSORT using the plant mode. Two of these had strong secretion signal predictions in both animal and fungi modes and in TargetP, whilst another isoform has a strong secretion signal prediction in these WoLF PSORT modes. This may indicate that some of these isoforms are targeted to the chloroplast via the endoplasmic reticulum (see below).

##### Targeting for Secretion

Proteins known to be in the Golgi, and which thus utilise the secretory pathway, were used as benchmarks to test the reliability of secretion signal prediction for *Euglena* proteins (Table 2). They were predominantly identified as being targeted for secretion by TargetP with a high level of confidence, especially using the nonplant algorithm, although mitochondrial targeting was predicted in some instances. WoLF PSORT predicted that these proteins were targeted to the plasma membrane, as they are integral membrane proteins. Some were predicted to also contain secretory signals with high confidence, but not all. One of the mannosyltransferases was predicted to target to the chloroplast using WoLF PSORT in plant mode.

##### Chloroplast Targeting

A selection of biochemical marker enzymes and components of the photosynthetic apparatus was used to test the ability of these programs to predict targeting to the plastids. TargetP predicted most of these proteins to be either mitochondrial or secreted (Table 3). The only exceptions were for one of the isoforms of fructose-bisphosphate aldolase, and one ribulose-bisphosphate carboxylase/oxygenase (small subunit) that were predicted to be targeted to the chloroplast after removal of the secretory signal peptide. WoLF PSORT on the other hand correctly predicted many soluble enzymes to be targeted to the chloroplast but predicted many of the integral membrane proteins, such as photosystem components, as being targeted to the plasma membrane.

The limitations of the chloroplast targeting prediction of TargetP have been reported before [29]. The predictive power of TargetP 1.1 is based on the presence of N-terminal presequences, including chloroplast transit peptide (cTP), mitochondrial targeting peptide (mTP), or secretory pathway signal peptide (SP) [29]. However, the structure of cTP is not well characterized, especially in *Euglena*, and the prediction performance of chloroplast targeted proteins was reported to be less accurate than that for mitochondria, with occasional poor discrimination between mTP and cTP [32]. This lack of discrimination is partly due to some proteins using the same targeting sequence for both chloroplasts and mitochondria [29]. Thus, using TargetP and WoLF PSORT to predict the location of proteins in *Euglena* might not cover all the possible protein transport systems.

**Figure 1:**
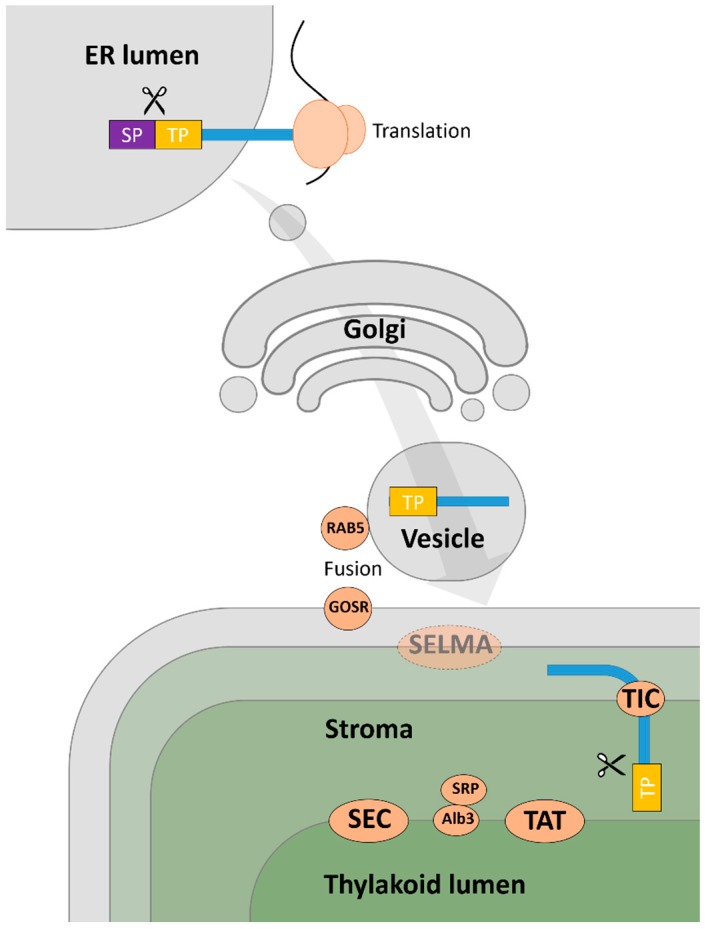
Protein transport into the secondary chloroplast of *Euglena*. Nuclear encoded chloroplast pre-proteins (blue strip) are synthesised into the lumen of the endoplasmic reticulum (ER) where the signal peptide (SP) is cleaved. Pre-proteins with transit peptides (TP) are subsequently transferred to the outermost chloroplast membrane through the Golgi body via vesicles. GOSR and RAB5 GTPase are proposed to mediate the fusion of the vesicle to the outermost membrane. After transport of proteins into the stroma, where the TP is removed, the mature protein can enter the thylakoid lumen via SEC, TAT, or Alb3/SRP pathway. This scheme only considers proteins possessing Class I and II targeting signals, as the transport of those with unclassified signals is not known [34].

Apart from the evident limitations of these algorithms as protein localisation prediction tools in *Euglena*, protein targeting into chloroplasts of *Euglena* is likely to be inherently complex. In contrast to plants, the chloroplast of *Euglena* evolved from the secondary endosymbiosis, which led to the chloroplast being surrounded by three membranes [25,26,33]. A recent study of the *E. gracilis* chloroplast proteome identified three classes of chloroplast pre-protein based on targeted signal analysis. Class I and II proteins possess a bipartite topogenic signal (BTS), with Class I proteins composed of a signal peptide (SP) followed by a stop-transfer signal (STS) and a transit peptide (TP), whilst Class II proteins contain only an SP and TP [31,34]. The third class of chloroplast proteins was referred to as unclassified, with no signal sequence detected in the proteins. The transport mechanism used to import proteins from this unclassified category into the plastid remains unknown [30]. The transport of *Euglena* Class I and II pre-proteins into the chloroplast involves the first step of co-translational transport into the endoplasmic reticulum (ER) lumen where the cleavage of the signal peptide occurs (Figure 1). The pre-proteins are subsequently transported to the chloroplasts from the Golgi body via vesicles, which then fuse with the outermost plastid membrane. However, the transport across the inner two membranes of the three-membrane-bound plastids in euglenophytes remains unclear [27,30,34]. The TOC/TIC-like pathway was believed to be involved in the inner membranes transport of the *Euglena* plastid due to the presence of plant-like targeting signal (TP) in the preproteins [35]. However, none of the TOC subunits have been detected in the transcriptome of *E. gracilis*, whereas homologues of several TIC subunits were identified [5]. A recent analysis of the structure of TP sequences in *E. gracilis* has suggested that it is possible for the TP to be recognised by the symbiont-derived ERAD-like machinery (SELMA) transport system, as is the case for diatoms [30,36].

It can be concluded that WoLF PSORT and TargetP have limitations with predicting cTPs and do not specifically include protein targeting to the secondary plastid. Predicting chloroplast protein targeting in *Euglena* is likely to require more specific databases or algorithms, since the evolution of the *Euglena* chloroplast is different from that of plants. In contrast, the prediction of mitochondria targeting with high reliability scores, when there is a high degree of agreement amongst the algorithms, can be informative. However, due to the false predictions of chloroplast proteins to other locations, the prediction results cannot be fully relied upon and need to be carefully evaluated in conjunction with evidence from enzymatic and biochemical analyses.

### 2.2. Pathway Localisation from Biochemical/Proteomic Information

#### 2.2.1. Central Metabolic Pathways of *Euglena*

The central metabolic pathways are essential to all organisms, providing the precursors for other peripheral pathways, especially metabolites with carbon backbones that are derived from carbohydrate metabolism. In addition, under non-photosynthetic conditions, these pathways have a major role in producing the energy and reducing power for the cell. Pathways of carbohydrate metabolism generally consist of glycolysis (Embden–Meyerhof–Parnas pathway), gluconeogenesis, the PPP, the Entner-Doudoroff (ED) pathway, and the TCA cycle. Notably, there is no evidence for the ED pathway in *Euglena*. Results for subcellular location predictions are available in Supplementary Table S1.

##### Glycolysis and Gluconeogenesis

The intracellular distribution of the glycolytic enzymes in *Euglena* has been studied using fractionation in aqueous and non-aqueous media. This approach showed that most of the glycolytic enzymes are in the cytosol and that several of them are present in both the chloroplast and the cytosol [37,38]. By using sucrose density gradient centrifugation, it was found that phosphofructokinase, pyruvate kinase, triosephosphate isomerase, and aldolase were present in the plastid fraction [39]. In addition, a recent proteomic study reported that several enzymes involved in glycolysis and gluconeogenesis were present in *Euglena* chloroplasts [30].

Hexose-Phosphorylating Enzymes. The activity of hexokinase (EC 2.7.1.1) was three times higher in *E. gracilis* grown on glucose than that on ethanol and acetate [40]. The activity of this enzyme in glucose media was also four times higher in heterotrophic cells than that in autotrophic cells [41]. *E. gracilis* was found to have glucokinase (EC 2.7.1.2) and fructokinase (EC 2.7.1.4) in different locations in both autotrophic and heterotrophic conditions. At 105,000 g separation, the glucokinase was present in the cell pellet while the fructokinase activity was only found in the supernatant [2,42]. Glucokinase is therefore concluded to be in organelles, whilst fructokinase is in the cytosol.

Phosphoglucoisomerase (EC 5.3.1.9). The activity of this enzyme was detected in *E. longa* [2,43], although, the subcellular location has not been reported. Strong targeting signals were not detected in the protein sequences.

6-Phosphofructokinase (ATP-PFK, EC 2.7.1.11) and Diphosphate--Fructose-6-Phosphate 1-Phosphotransferase (PPi-PFK, EC 2.7.1.90). In *E. gracilis*, ATP-PFK was reported to be located in both chloroplasts and the cytosol [39], while PPi-PFK was reported exclusively in the cytosol. During cell growth, the activity of PPi-PFK was 10–30 times higher than the activity of ATP-PFK [44]. No strong targeting signals were detected in these protein sequences.

Fructose Bisphosphate Aldolase (EC 4.1.2.13). There are two classes of aldolase found in *Euglena*: Class I is located in the chloroplast and proplastid, and Class II is located in the cytosol [45]. Class I enzyme peptides were detected in the chloroplast proteome [30] and the Class II cytosolic enzyme was shown to be more active when the *E. gracilis* culture was grown in the dark and is presumed to play the main role in heterotrophic glycolysis and gluconeogenesis [46]. One isoform has no strong targeting signal, whilst two have plastid targeting and one has a strong mitochondrial targeting sequence.

Glyceraldehyde 3-phosphate Dehydrogenase (G3P) Dehydrogenase (EC 1.2.1.12). *E. gracilis* contains both NAD-linked and NADP-linked G3P dehydrogenase, which are found in different subcellular locations [45,47]. The NAD-linked enzyme showed higher activity in heterotrophic conditions and was located in the cytosol. On the other hand, the NADP-linked enzyme was shown to be located in chloroplasts and had higher activity in autotrophic cells [48]. Only the NADP-linked enzyme was detected in the proteome of *E. gracilis* chloroplasts [30].

Triosephosphate Isomerase (EC 5.3.1.1). As with fructose bisphosphate aldolase, two types of the isomerase were identified in *E. gracilis* using enzymatic activity profiling [47]. Type A triosephosphate isomerase was reported to function in the chloroplasts and proplastids of *E. gracilis*, while type B enzymes were located in the cytosol [49]. Sequences matching triosephosphate isomerase could also be detected in the *E. gracilis* chloroplast proteome [30].

Phosphoglycerate Kinase (EC 2.7.2.3)/Phosphoglycerate Mutase (EC 5.4.2.11). The activity of phosphoglycerate kinase was reported in isolated *E. gracilis* chloroplasts [50] and the enzyme was detected in the *E. gracilis* chloroplast proteome [30], although the presence in other subcellular locations has not been investigated. No specific studies of the activity of phosphoglycerate mutase have been reported in *Euglena*. However, the enzyme was recently reported to be present in the *E. gracilis* chloroplast proteome [30]. WoLF PSORT identifies a strong chloroplast targeting sequence on one isoform, with the other three isoforms is predicted to remain in the cytosol.

Enolase (EC 4.2.1.11). The activity of enolase was previously detected in *E. gracilis* but the subcellular location was not described [38,51]. N-terminal targeting peptide analysis of cDNA clones of *E. gracilis* suggested that enolase could be present in both the cytosol and the chloroplast [52]. However, as shown in Section 2.1.3, it is difficult to predict protein targeting into the chloroplasts of *Euglena* and, furthermore, enolase was not found in the chloroplast proteome of *E. gracilis* [30].

Pyruvate Kinase (EC 2.7.1.40). The activity of pyruvate kinase in *E. gracilis* was shown to be highly active in cultures grown on glucose [53]. This enzyme was reported to be located in both proplastids and the cytosol of *E. gracilis*, however, the activity of this enzyme was not detected in the mature chloroplast [39]. WoLF PSORT predicts plastid targeting sequence in two isoforms with very low confidence, whilst one of these has mitochondrial targeting with slightly more confidence, highlighting the challenging nature of predicting subcellular locations.

Fructose-1,6-Bisphosphatase (EC 3.1.3.11). Fructose-1,6-bisphosphatase is involved in gluconeogenesis and has been reported from *Euglena* [39,44]. The cytosolic fructose-1,6-bisphosphatase in *E. gracilis* was detected and characterized [54]. Recently, the enzyme was reported in the *E. gracilis* chloroplast proteome [30], where it is presumably involved in the Calvin cycle. One isoform is predicted not to contain a targeting signal, but the other four are predicted to be variously targeted to the chloroplast, for secretion, or to the plasma membrane, possibly indicating that they all pass through the secretory system to the chloroplast.

##### Pentose Phosphate Pathway

Oxidative Phase. In contrast to higher plants and green algae, all the enzymes of the oxidative arm of the pentose phosphate pathway in *E. gracilis* were reported to be present in the cytosol, but not the chloroplast. Using non-aqueous fractionation, it was found that two dehydrogenases of the oxidative pentose phosphate pathway were absent from the *E. gracilis* plastid [37]. In separate studies, the activity of 6-phosphogluconate dehydrogenase (EC 1.1.1.44) was confirmed to be in the cytosol [38], and glucose-6-phosphate dehydrogenase (EC 1.1.1.49) was reported to be located in the cytosol [2,38,55,56,57] and has been used as a cytosolic marker enzyme [58]. Although a single glucose-6-phosphate dehydrogenase was detected in the chloroplast proteome, this fraction was reported to be moderately contaminated with protein from other organelles [30] and thus, subcellular location of the enzyme will need further investigation to confirm its location. This enzyme is specific for NADP in *Euglena* and induced by glucose, with low activity detected under heterotrophic growth in the absence of glucose [53]. There has been no specific study of *Euglena* 6-phosphogluconolactonase (EC 3.1.1.31).

Non-Oxidative Phase. All the enzymes involved in the non-oxidative section of the pentose phosphate pathway have been detected in *Euglena* and most of the enzymes were reported to localize to the chloroplast [2,30]. The activity of ribose 5-phosphate isomerase (EC 5.3.1.6) was reported in isolated *E. gracilis* chloroplasts [59]. The subcellular location of pentose-5-phosphate-3-epimerase (EC 5.1.3.1) has not been reported, although the activity of this enzyme was detected in heterotrophic, autotrophic, and mixotrophic growth conditions, along with the activity of transketolase (EC 2.2.1.1) [60] and transaldolase (EC 2.2.1.2) [47]. Non-aqueous separation techniques showed the presence of transaldolase in *Euglena* chloroplasts and proplastids [39].

Notably, there are two isoforms of each enzyme of the non-oxidative PPP in the *E. gracilis* transcriptome, except transketolase which has three. For three of these enzymes, only one isoform was identified in the chloroplast proteome [30], whereas neither isozyme of transaldolase could be detected. This suggests that the other isoforms are present in another location within the cell and the lack of any detectable targeting signal indicates this is likely to be the cytosol. However, extensive study of this pathway has not been reported and further investigation would be needed to confirm the operation of the pathway in the cytosol.

##### Anaplerotic Pathway: Dicarboxylic Acid Bypass

Malate dehydrogenase (NADP-specific oxaloacetate-decarboxylating, EC 1.1.1.40) in *Euglena* is located in the cytosol but not in mitochondria, and is specific for NADP and l-malate [2]. The NAD-specific malate dehydrogenase (decarboxylating, EC 1.1.1.39) can only be detected in *E. gracilis* cultured with d-malate [61]. Recently, a proteomic study detected malate dehydrogenase (NADP-specific) in *E. gracilis* chloroplasts [30]. The activity of this enzyme varied widely with light and carbon sources, and has 55 times greater activity in heterotrophic cells than in autotrophic cells. This result suggests a physiological role in *Euglena* for these enzymes in providing NADPH for cytosolic fatty-acid synthesis in the dark [62,63].

Phosphoenolpyruvate carboxylase (PEP carboxylase, EC 4.1.1.31) was shown to have multiple isozymes which were active in different light conditions. It has been reported that PEP carboxylase functions for CO_2_ fixation in *E. gracilis* grown in the dark and under CO_2_ limited conditions [64,65]. The activity of phospho*enol*pyruvate carboxykinase (PEP carboxykinase, EC 4.1.1.32) in *E. gracilis* is specific for GTP rather than ATP [66]. PEP carboxylase and PEP carboxykinase are discrete, separate enzymes in *E. gracilis* [67]. PEP carboxykinase was reported to be located exclusively in the cytosol and the enzyme could not be detected in cells grown under autotrophic conditions [68]. One isoform is predicted to be localised in the chloroplast by WoLF PSORT with a high degree of confidence, but the locations of the other two isoforms are not predicted confidently. In addition, the activity of PEP carboxykinase was detected in *E. gracilis* cultured with acetate or ethanol, but not with glucose [62]. Pyruvate carboxylase (EC 6.4.1.1) was also reported to be located in the cytosol [69]. The activity of this enzyme was found in cells grown under heterotrophic culture fed with glucose, but not with acetate or in autotrophic cells [2].

##### TCA Cycle

The reactions of the TCA cycle occur in the mitochondria of *Euglena* in common with all other eukaryotic organisms [2]. Most of the enzymes involved in the TCA cycle are predicted to target to the mitochondria with high reliability (Table S2), in line with previous studies on the localisation of the TCA cycle.

Pyruvate Dehydrogenase (NAD complex 1.2.4.1, NADP+ EC 1.2.1.51). In *E. gracilis* the conventional NAD^+^ pyruvate dehydrogenase complex only contributes around 1% of the activity and instead an NADP^+^-dependent pyruvate dehydrogenase is used to produce the majority of the acetyl-CoA from pyruvate [70]. This latter enzyme has been detected in the mitochondrial fractions of *E. gracilis* [71,72,73] and all three component polypeptides are predicted to be targeted to the mitochondria. The activity of the NAD complex has not been localised.

Citrate Synthase (EC 4.1.3.7). Citrate synthase activity was detected in both particulate and soluble fractions from bleached *E. gracilis* [38], indicating that the enzyme is located in cytosol and other cell compartments. Testing the activity of this enzyme from different organelle suspensions showed the presence of this enzyme in both mitochondria and microbodies (glyoxysome-like particles) [74,75]. Only one of the four isoforms is predicted to be targeted to mitochondria.

Aconitase (EC 4.2.1.3). The activity of aconitase was detected in *E. gracilis* [76,77]. However, the subcellular location of this enzyme has apparently never been investigated and only one of the two isoforms is predicted to be targeted to mitochondria.

Isocitrate Dehydrogenase (NAD-specific EC 1.1.1.41, NADP-specific EC 1.1.1.42). NAD- and NADP-specific isozymes of isocitrate dehydrogenase have been characterised from *Euglena*. The activity of NAD-specific isocitrate dehydrogenase was detected in mitochondria and cytosol of *E. longa* [38,43]. The NAD-specific isozyme was detected solely in the mitochondria of the streptomycin-bleached *E. gracilis* [75,78,79]. The NADP-specific isozyme was reported in both mitochondria and the cytosol, with the activity of the mitochondrial enzyme being about 25% of that in the cytosol [75,79].

2-Oxoglutarate Decarboxylase (EC 4.1.1.71). *E. gracilis* contains a 2-oxoglutarate decarboxylase that is dependent on thiamine pyrophosphate, in contrast to the more common CoA-dependent 2-oxoglutarate dehydrogenase complex, which was not detected [80]. The thiamine pyrophosphate dependent activity which coverts 2-oxoglutarate to succinic semialdehyde is located solely in mitochondria [81].

Succinic Semialdehyde Dehydrogenase (EC 1.2.1.16). NAD- and NADP-specific succinate semialdehyde dehydrogenase were detected in *E. gracilis* and reported to be in the mitochondria [73,82]. Three isoforms are predicted to be located in the mitochondria, whilst the remaining isoform is not predicted to have a targeting sequence.

Succinate Dehydrogenase (EC 1.3.5.1). As with other eukaryotes, the succinate dehydrogenase in *E. gracilis* is tightly bound to the inner membrane of mitochondria and has been used as a marker enzyme for mitochondria in *Euglena* [83]. [58,74,75,78]. It is predicted to be associated with the plasma membrane by WoLF PSORT, in line with the integral membrane nature of the protein.

Fumarase (EC 4.2.1.2). Using cell fractionation and enzyme activity assays, fumarase is routinely detected solely in *E. gracilis* mitochondria [39,74,75,78] and is commonly used as a soluble mitochondrial marker enzyme [83].

Malate Dehydrogenase (EC 1.1.1.37). In *E. gracilis,* malate dehydrogenase is found in both mitochondria and the cytosol. The cytosolic enzyme had three times higher activity in heterotrophically grown cells than in photoautotrophic cells, whereas the activity of the mitochondrial isoform was largely uninfluenced by variation in growth conditions [62]. *E. gracilis* contains two forms of malate dehydrogenase, NAD-linked and NADP-linked isozymes. Unlike in higher plants, where the NADP-linked malate dehydrogenase is present exclusively in chloroplasts, in *E. gracilis* the majority (81–91%) of both NAD-linked and NADP-linked activity were located in the cytosol with a smaller proportion (13–16%) found in mitochondria. The activity of the NAD-linked isozyme was reported to be about three times higher than that of the NADP-dependent isozyme [84,85].

##### Glyoxylate Cycle

The glyoxylate cycle is a modified form of the TCA cycle that is found in plants, bacteria, protists and fungi. The cycle has an important role in provision of precursors for gluconeogenesis and allows the cell to use other respiratory substrates when sugars are not available [86]. The subcellular location of the glyoxylate cycle in *Euglena* under different conditions is poorly defined, with studies suggesting that the cycle operates in either mitochondria or discrete microbodies (glyoxysome-like particles). Notably, the presence of microbodies in *E. gracilis* was reported to vary under different conditions [87]. Following cell fractionation on sucrose density gradients, the activities of isocitrate lyase (EC 4.1.3.1) and malate synthase (EC 2.3.3.9), enzymes unique to the glyoxylate cycle, were found in the microbody fraction of *E. gracilis* grown on acetate [75,78]. In contrast, using similar cell fractionation techniques and immunocytochemical analysis, both isocitrate lyase and malate synthase were localised to mitochondria in *E. gracilis* grown on ethanol in which microbodies could not be detected [88].

##### C2 Metabolism

Ethanol, which can readily diffuse into the cell, is first oxidized to acetaldehyde by alcohol dehydrogenase (EC 1.1.1.1), and the product is then oxidised by acetaldehyde dehydrogenase (EC 1.2.1.10) to produce acetate. Both enzymes are found in *E. gracilis* mitochondria [89,90,91]. Acetate is taken up either by simple diffusion or active transport through monocarboxylate transporters and is then converted to acetyl-CoA by acetyl-CoA synthetase (EC 6.2.1.1), also located in *E. gracilis* mitochondria [92], and then metabolized through the TCA cycle or channelled into the glyoxylate cycle.

#### 2.2.2. Subcellular Locations of Biomass Production

The composition of *Euglena* biomass is similar to that of many organisms, with storage carbohydrates, proteins and lipids predominating. The amounts of the different components varies substantially depending on the growth conditions, from almost 10% dry weight wax esters [93] under anaerobic growth to over 80% paramylon under aerobic conditions [94].

##### Carbohydrate Biosynthesis

Unlike most other photosynthetic organisms, such as plants and green algae, *Euglena* stores carbohydrate in the form of a crystalline β-1,3-glucan, called paramylon, instead of starch, and the soluble disaccharide trehalose, instead of sucrose. *Euglena* has a wide range of enzymes involved in carbohydrate metabolism but it is difficult to predict their substrates and products from sequence alone [95].

Paramylon. Paramylon is synthesized from UDP-glucose [96] using the membrane bound paramylon synthetase (beta-1,3-glucan beta-glucosyltransferase, EC 2.4.1.34) that was identified in the *E. gracilis* mitochondrial fraction by measuring activity following differential centrifugation [97] and the genes identified in the transcriptome [98]. Based on transmission electron microscopy, paramylon was synthesised in vesiculated mitochondrial related membrane complexes (chondriomes). The matrix of these vesicles was dense with paramylon granules and extended into the cytosol. The vesicles developed, resulting in the membrane-bound paramylon grains found in the cytosol [41,99,100]. The endo-1,3-β-glucanases (EC 3.2.1.6 and EC 3.2.1.39), exo-1,3-β-glucanases (EC 3.2.1.58), and 1,3-β-glucan phosphorylases (EC 2.4.1.97) involved in glucan metabolism have been characterized [101,102,103], though the subcellular locations of these enzymes have not been defined. Some of these are predicted to be membrane associated or chloroplast localised.

Trehalose. In *Euglena gracilis*, trehalose synthesis was reported to have a role in the acclimation to osmotic stress [104,105]. Trehalose biosynthesis involves a two-step process through the sequential action of trehalose-phosphate synthase (TPS, EC 2.4.1.15) and trehalose-phosphate phosphatase (TPP, EC 3.1.3.12). It was found that the activities of TPS and TPP could not be separated and so a TPS/TPP enzyme complex of about 250 kDa was suggested to be responsible for trehalose synthesis in *E. gracilis* [106]. In *Arabidopsis*, the bulk of the TPP was reported to be cytosolic [107,108]. However, the subcellular localisation of the TPS/TPP complex in *Euglena* has not been investigated. Analysis of the chloroplast proteome of *E. gracilis* [30] shows no evidence of the TPS and TPP suggesting it is more likely that the TPS/TPP complex is located in the cytosol (or conceivably mitochondria) rather than in chloroplasts. There is no strong targeting signal predicted for this enzyme, supporting the putative cytosolic location.

##### Amino Acid Biosynthesis

The pathways of amino acid biosynthesis in *Euglena* have been poorly investigated, especially with regard to their subcellular localisation. The recent evidence from the proteomic analysis of *Euglena* chloroplasts suggested that their capacity for synthesis of amino acids is extremely limited, in contrast to plant and algal chloroplasts, which are the major subcellular sites for synthesis of various amino acids [30]. Here we present a summary of the likely subcellular localisation of amino acid biosynthesis in *Euglena*.

Glycine and Serine (Glycolate Pathway Associated). Glycine and serine are synthesised from glyoxylate, an intermediate of photorespiration and gluconeogenesis. Glycolate dehydrogenase (EC 1.1.99.14), the starting enzyme of the glycolate pathway, was reported to be located in both mitochondria and microbodies in *E. gracilis* [78]. Glutamate:glyoxylate aminotransferase (EC 2.6.1.4), which adds the amino group to form glycine [109], is found in mitochondria, the cytosol and microbodies [78,110]. A small proportion of the glyoxylate is converted to glycine by glutamate:glyxoylate aminotransferase in mitochondria, and the majority is split into CO_2_ and formate. As in higher plants, the formate is then used to produce serine through condensation with glycine [111,112]. Folate coenzymes, which are involved in this C1 transfer, were reported to be located largely in the cytosol [79]. Glycine can also be produced through the cleavage of threonine by threonine aldolase (EC 4.1.2.5/48) [113], though the subcellular location of this activity has not been reported. The enzymes involved in serine biosynthesis from 3-phosphoglycerate have not been studied in detail in *Euglena*. However, recently, phosphoserine phosphatase was identified in the *E. gracilis* chloroplast proteome, indicating the possibility of a plastidic serine biosynthesis pathway [30].

Methionine, Cysteine, and Threonine. The activity of cobalamin-dependent methionine synthase (EC 2.1.1.13), producing methionine from N^5^-methyltetrahydrofolate and homocysteine, was reported to be distributed between the cytosol (68.9%), chloroplast (18.4%) and mitochondria (9.5%) of phototrophic cells. The more stable, Mg-dependent, variant was reported to be found only in the cytosol [114]. Cysteine synthesis in *Euglena* has not been investigated in detail and the subcellular localisations of the enzymes associated with this pathway have not been elucidated. Two enzymes involved in the synthesis of cysteine (serine O-acetyltransferase and cysteine synthase) were reported in the *E. gracilis* transcriptome [113] and isoform A of cysteine synthase was detected in the *E. gracilis* chloroplast proteome [30]. Threonine is synthesized from aspartate via homoserine. Five enzymes involved in threonine biosynthesis in *E. gracilis* were reported to be expressed in different growth conditions [113]. However, the localisations of the enzymes involved in the synthesis pathway have not been elucidated.

Aromatic Amino Acids (Phenylalanine, Tyrosine, and Tryptophan). Chorismate, the precursor to aromatic amino acids, is synthesised from d-erythrose 4-phosphate and phosphoenolpyruvate by the shikimate pathway in seven steps. Five reactions can be catalysed either by separate enzymes, as in plants [115], or by a pentafunctional enzyme, as in fungi [116]. There is evidence for both of these in the *E. gracilis* transcriptome [27].

In green algal and plant cells, the aromatic amino acids are produced exclusively in the plastid but the protein analysis of isolated organelles of *E. gracilis* suggests that the shikimate pathway occurs in both the chloroplast and cytosol [117]. The preferred pathway depends on the growth conditions, with the cytosolic pathway used in the dark and the plastidic pathway in the light [117,118].

Chorismate is then converted into tyrosine and phenylalanine, via prephenate by dehydration, dehydrogenation, and transamination. The enzymes catalysing these reactions are present in *E. gracilis* as unusual domain fusions, also found in thermophilic bacteria [16]. Tryptophan is synthesised from chorismate by a series of reactions via anthranilate. In *E. gracilis* all four of these reactions are carried out by a unique fusion protein rather than a series of separate enzymes, as in other organisms [11,113].

Together the data suggest that aromatic amino acid biosynthesis in *Euglena* is carried out by a combination of plant-, bacterial-, and fungal-like enzymes, as well as unique proteins. The evidence suggests that these pathways are not exclusively located in the plastid, unlike in plants, supporting the dispensability of the plastid for their biosynthesis.

Branched-Chain Amino Acids (Valine, Isoleucine, and Leucine). Pyruvate and α-ketobutyrate are the precursors for valine, leucine and isoleucine biosynthesis in *Euglena*, as in other organisms [119]. In *E. gracilis*, α-ketobutyrate is synthesized by the action of two threonine dehydratases (EC 4.3.1.19 and EC 4.3.1.17) that are located in the cytosol [120]. The subsequent steps are catalysed by acetolactate synthase, dihydroxy-acid reductoisomerase, and branched-amino-acid aminotransferase, all of which are located in the mitochondria [119], suggesting the biosynthesis of branched-chain amino acids is located in mitochondria.

Arginine and Proline. Arginine is synthesised by the sequential transfer of nitrogen onto glutamate semialdehyde. Arginine biosynthesis is likely to occur mostly in the cytosol in *Euglena*, as the majority of ornithine carbomyltransferase is located in the cytosol and smaller portion in mitochondria [2]. Arginine metabolism follows the arginine dihydrolase pathway in which arginine is converted into citrulline and then ornithine, which occurs in the mitochondria [121]. Proline synthesis in *Euglena* has not been investigated. However, proline metabolism is tightly associated with arginine metabolism as ornithine is the precursor for proline synthesis [122], suggesting that synthesis is likely to be located in the cytosol or mitochondria.

Lysine. Bacteria, plants and algae synthesize lysine via the diaminopimelate (DAP) pathway, using aspartate and pyruvate as the precursors. On the other hand, fungi synthesize lysine through the α-aminoadipate (AAA) pathway, which uses 2-oxoglutarate and acetyl-CoA [123,124]. Several enzymes involved in AAA pathway were detected in *Euglena*, including homocitrate synthase (EC 2.3.3.14), homoaconitate hydratase (EC 4.2.1.36) and homoisocitrate dehydrogenase (EC 1.1.1.87) [113]. However, the subcellular location of the AAA pathway has not been reported.

Histidine. Histidinol dehydrogenase, the enzyme catalysing the final step of histidine biosynthesis, has been detected in *E. gracilis* [113,125]. No other enzyme involved in this process was detected and the subcellular localisation of the enzymes involved in histidine biosynthesis have not been investigated.

Glutamate, Glutamine, Alanine, Aspartate, and Asparagine. Aminotransferases and dehydrogenases play the main role in the synthesis of glutamate, alanine, and aspartate from organic acids. For glutamate, the aspartate aminotransferase (glutamate: oxaloacetate aminotransferase) is present in mitochondria, chloroplasts, microbodies, and cytosol, and was shown to be more active in dark growth conditions [74,78]. NADP-specific glutamate dehydrogenase was reported to be located solely in the cytosol of *E. gracilis*, instead of the mitochondria as in other organisms [126]. Similarly, glutamate synthase was reported to be localised to the cytosol in both wild-type and streptomycin-bleached *E. gracilis* strains [127]. Glutamine is synthesized from glutamate using glutamine synthetase, but the properties of this enzyme have not been studied in *Euglena* [128]. Asparagine synthetase, the enzyme that converts aspartate to asparagine, has not been reported from *Euglena*. The activities of alanine aminotransferase and alanine dehydrogenase were detected in *E. gracilis*, but the localisation of these enzymes has not been described [2,115,116].

Tetrapyrrole Biosynthesis. Tetrapyrrole, the core of heme and chlorophyll, is synthesised from δ-aminolevulinic acid (ALA). Heterotrophs tend to synthesize ALA from glycine and succinyl-CoA via the Shemin pathway in the mitochondia [129], whilst photoautotrophs make ALA from glutamate in the C5 pathway, located in the chloroplast [130]. *E. gracilis* is known to utilise both routes [131], and the transcriptome shows a bacterial-derived Shemin pathway and a green algae-related C5 pathway, presumably obtained with the chloroplast [16]. These have been identified in the mitochondria and chloroplasts of *E. gracilis* respectively [132]. This again supports the multiple locations of core metabolic pathways that are plastid localised in other photosynthetic organisms.

##### Lipid Biosynthesis

The subcellular locations of the enzymes involved in lipid metabolism in *Euglena* are poorly investigated. As in other organisms *Euglena* produces the lipid building block malonyl-CoA from CO_2_ and acetyl-CoA using acetyl-CoA carboxylase, which forms a multienzyme complex with phosphoenolpyruvate carboxylase and malate dehydrogenase in the cytosol [133]. Malonyl-CoA is then used to synthesise fatty acid using fatty acid synthases (FAS), of which three types have been reported in *E. gracilis*. FAS I and FAS III were reported to function in heterotrophic growth conditions. The properties of FAS III has not been investigated in detail. The structure of FAS I is similar to yeast and mammalian enzymes, and was located in cytosol [2]. On the other hand, FAS II resembles the plant and bacterial enzymes, and is located in the chloroplasts of *E. gracilis* [134]. In addition to these three types of FAS, a fatty acid biosynthesis system was found in the mitochondria of *Euglena* and is involved in wax-ester synthesis [134].

## 3. Discussion

**Figure 2:**
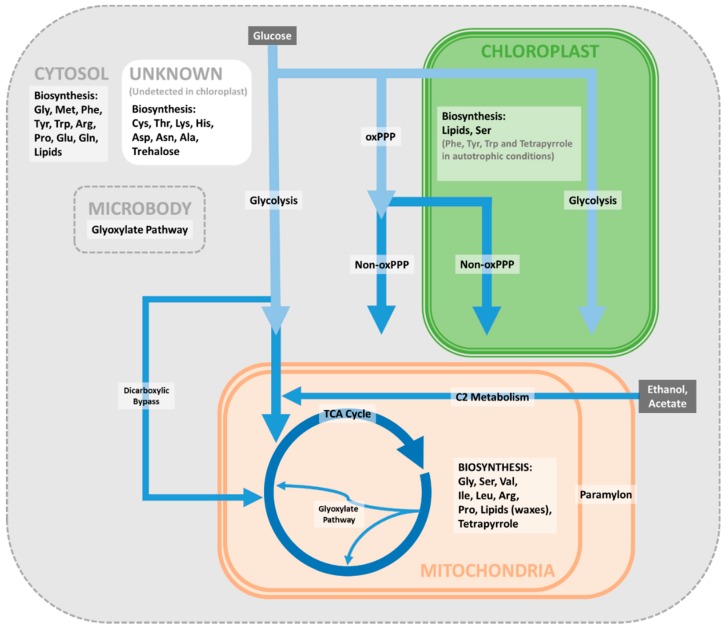
Proposed distribution of central metabolic pathways in *Euglena*. Abbreviations: oxPPP—oxidative pentose phosphate pathway; Non-oxPPP—non-oxidative pentose phosphate pathway.

By combining multiple strands of evidence, including biochemical, proteomic, and bioinformatic data, we propose a model for the subcellular localisation of the reactions of the network of central carbon metabolism in *E. gracilis* (Figure 2). Many of these pathways are found in similar subcellular locations to those in other, well-characterised organisms. Glycolysis, which catalyses the initial breakdown of sugars produced by photosynthesis or absorbed from the medium, is present in the cytosol and plastids, as commonly found in green plants. The products of this pathway feed into the TCA cycle, which is mitochondrial, as in other eukaryotes. The enzymes commonly associated with microbodies in higher plants are additionally present in the mitochondria, and it is often difficult to separate these two groups of organelles in *Euglena*. The site of synthesis of many amino acids is unclear, though several appear to be synthesised in the mitochondria from TCA cycle intermediates. Lipids can be made in several cellular compartments, though for different purposes, such as the mitochondrial lipids which are directed towards wax ester biosynthesis and plastid lipids that are used to make photosynthetic glycolipids.

However, the locations of many metabolic processes in *Euglena* differ substantially from those found in other photosynthetic organisms. For instance, in *Euglena* the complete PPP is present in the cytosol, with a duplicated non-oxidative phase present in the plastid. A plant-like pathway for aromatic amino acid biosynthesis is present in the plastids [117]. However, unlike plants, in *Euglena* an additional pathway, similar to that found in fungi, is located in the cytosol. Tetrapyrroles, essential prosthetic groups of both the respiratory and photosynthetic electron transport chain proteins, are synthesised in both the chloroplast and mitochondria in *Euglena*.

Overall, these results indicate that, aside from the reactions of photosynthesis, all the metabolic pathways found in the *Euglena* plastid are also found elsewhere in the cell. This includes the biosynthesis of isoprenoids, for which two pathways are found in other plastid-containing organisms, the methylerythritol phosphate pathway found in the plastids and the mevalonate pathway in the cytosol. Although we have not found evidence for the location of these pathways in *Euglena*, the methylerythritol phosphate pathway only contributes to carotenoid biosynthesis in *E. gracilis*, and phytol is instead made by the mevalonate pathway [135], unlike in other studied organisms. The unusual and well-established ability of *E. gracilis* to survive on a simple carbon source when their chloroplasts have been destroyed can be rationalised from the subcellular localisation and duplication of these various critical pathways.

The complicated evolutionary history of *Euglena* means it is not trivial to predict the likely subcellular locations of the various metabolic pathways, or to decide whether the pathways will be similar to those in free-living heterotrophs, or plants, or be entirely different. Precise information is missing for some biosynthetic pathways and the lack of understanding of *Euglena* chloroplast protein targeting restricts the prediction of the subcellular location of some *Euglena* proteins. Despite these limitations, overall, the model is similar to plants and green algae, but has some important differences. The development of this model will lead to the ability to predict the metabolic phenotypes of *Euglena* under various growth conditions.

## 4. Conclusions

The subcellular compartmentation of metabolism has been intensively studied in yeast and in plants. For many, more distantly related organisms, most information is typically inferred by extrapolation from these thoroughly examined species. Drawing on a range of *Euglena* biochemical and proteomic data, we propose a model for the organisation of central metabolism in *E. gracilis*. These analyses reveal unique features of this alga that diverge significantly from expectations derived from well-studied organisms. The most striking difference in *Euglena* is the presence of extra activities of the enzymes of various biosynthetic pathways solely present in the plastids of plants, contributing to the ability of *Euglena* to lose its plastid entirely and survive on simple carbon sources. We propose that this is due to the requirement of the heterotrophic ancestor to synthesise all necessary cellular components before the acquisition of the secondary plastid. In this context, it seems likely that the plastid pathways are replicating pathways that were originally present in the euglenid progenitor.

## 5. Materials and Methods

### 5.1. Identification of Euglena Enzymes

The transcriptome of *E. gracilis* was searched for the target proteins using BLASTP with templates that were selected from the corresponding enzymes from other organisms represented in the NCBI databases. Identified *E. gracilis* transcripts were then used as templates to interrogate the NCBI databases, to confirm the correct identification of the proteins. The presence of a spliced leader was confirmed in 39% of all of these sequences, in the range previously reported in Euglena transcriptomes [16,17], by searching for a 10 bp sequence (TTTTTTTTCG or ATTTTTTTTC) at the 5′ end of the transcript.

### 5.2. ProteinTargeting Prediction for Euglena

**Figure 3:**
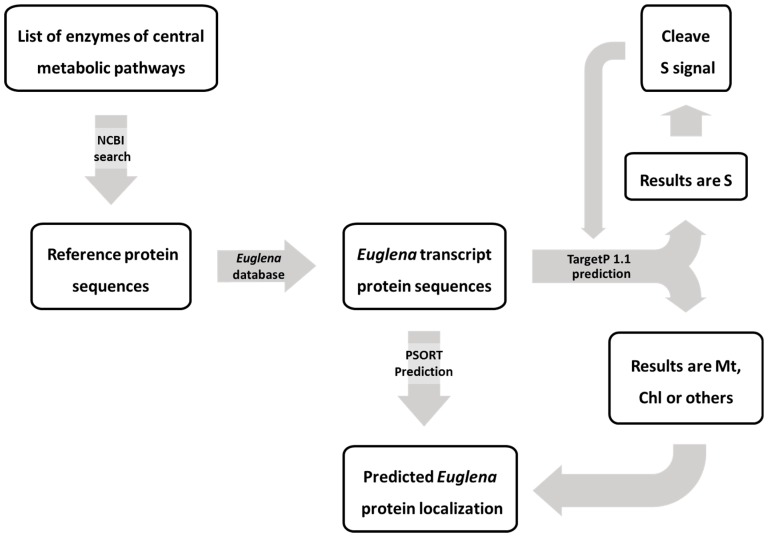
Subcellular location prediction workflow for *Euglena* proteins. Abbreviations: Mt—mitochondria; Chl—chloroplast; others—cytosol; S—secretory pathway.

A selection of proteins known to be localized to the chloroplast, mitochondria, Golgi or cytosol [2,136,137] were used to validate the use of WoLF PSORT [28] and TargetP 1.1 [29] (Table 1, Table 2 and Table 3). For proteins predicted to be secreted by TargetP using the plant search parameters, the signal sequence was removed, using the algorithms predicted cleavage site (Figure 3). The remaining sequence was then reanalysed to identify any alterations in targeting and potentially unveil a chloroplast targeting sequence. WoLF PSORT did not predict any secreted proteins using the plant search parameter. Results for metabolic pathway components are available in Supplementary Table S1.

## Figures and Tables

**Table 1:** Subcellular location prediction of *E. gracilis* mitochondria marker proteins.

Marker Enzyme Name	EC Number	EG Transcript	WoLF PSORT Plant			WoLF PSORT Animal			WoLF PSORT Fungi			TargetP Plant	TargetP Nonplant	TargetP, Signal Cut
**Fumarase**	**4.2.1.2**	**4764**	**Chl: 8**	-		**Mt: 27.5**	Cyt_Mt: 17	-	Mt: 26	-	-	Mt: 1	Mt: 2	-
Succinic semialdehyde dehydrogenase	1.2.1.16	**7552**	Cyt: 11	-		Cyt: 24	-	-	Cyt: 22	-	-	Cyt: 2	Cyt: 1	-
		5575	Mt: 10.5	Chl_Mt: 7.5		Mt: 28.5	Cyt_Mt: 16	-	Mt: 22.5	Cyt_Mt: 14	-	Mt: 1	Mt	-
		**3780**	Chl: 9	-		Mt: 10.5	Cyt_Mt: 10.3	Cyt: 9	Mt: 16	Cyt: 7.5	-	Mt: 5	Mt: 4	-
		**5750**	Mt: 12.5	Chl_Mt: 7.5		Mt: 27.5	Cyt_Mt: 16.5	-	Mt: 25.5	Cyt_Mt: 14	-	Mt: 3	Mt: 2	-
Isocitrate dehydrogenase	1.1.1.41, 1.1.1.42	6875	Cyt: 7	-		Cyt: 17	Mt: 15	-	Cyt: 22.5	Cyt_Nu: 12	-	Cyt: 3	Cyt: 2	-
		**10115**	Chl_Mt: 7.5	Mt: 7	Chl: 6	Mt: 29	Cyt_Mt: 16.5	-	Mt: 22	-	-	Mt: 1	Mt: 1	-
		10872	Mt: 10	-		Mt: 28	-	-	Mt: 26.5	Cyt_Mt: 14	-	Mt: 3	Mt: 5	-
		**10979**	-	-		Mt: 24	Cyt_Mt: 15.3	Mt_Per: 12.8	Per: 15	Cyt: 7.5	-	Mt: 2	Mt: 2	-
NADPH:glyoxylate reductase	1.1.1.79	**5154**	Mt: 14	-		Mt: 24	Cyt: 6	-	Mt: 14.5	CytMt:14	Cyt: 12.5	Mt: 3	Mt: 5	-
		13699	Cyt: 7	-		Mt: 27	-	-	Cyt: 12.5	Mt: 10	Cyt_Nu: 7	Cyt: 5	Sec: 2	-

Transcript numbers in bold indicate the presence of the splice leader sequence. PSORT score is the discriminant score, with larger scores having a higher probability. Scores below 5 are not reported. TargetP score is the reliability class is rated from 1 to 5 (1 is the strongest prediction and 5 is the weakest). Chl—chloroplast (green); Cyt—cytosol (grey); E.R.—endoplasmic reticulum (Blue); Mt—mitochondria (orange); Nu—nuclear; Per—peroxisome; PM—plasma membrane (yellow); Sec—secreted or extra cellular (blue). Strength of colour indicates score.

**Table 2:** Subcellular location prediction of *E. gracilis* cytosol (grey) and Golgi (blue) marker proteins.

Marker Enzyme Name	EC Number	EG Transcript	WoLF PSORT Plant		WoLF PSORT Animal			WoLF PSORT Fungi			TargetP Plant	TargetP Nonplant	TargetP, Signal Cut
Glutamate dehydrogenase	1.4.1.2	11196	Cyt: 8	Chl: 6	Cyt_Nu: 15	Nu: 8.5	-	Cyt: 16	Cyt_Nu: 9.5	Cysk:7	Cyt: 2	Cyt: 2	-
		10682	Cyt: 8	-	Cyt: 21	Cyt_Nu: 16	Nu: 9	Cyt: 12.5	Mt: 8	Cyt_Nu: 8	Cyt: 4	Cyt: 3	-
		7536	Cyt: 11	-	Cyt: 16.5	Cyt_Mt: 13.6	Cyt_Nu: 10.3	Mt: 15	Cyt: 7.5	-	Mt: 5	Cyt: 4	-
		791	Cyt: 6	-	Cyt: 12	Nu: 10	-	Cyt: 11.5	Cyt_Nu: 7.5	Mt: 7	Cyt: 4	Cyt: 5	-
NAD-lactate dehydrogenase	1.1.1.27	11571	-	-	Mt: 10	Cyt: 7	Cyt_Nu: 7	Mt: 19	Cyt: 8	-	Cyt: 3	Cyt: 5	-
thiosulfate sulfurtransferase	2.8.1.1	**32721**	Cyt: 8.5	-	Cyt: 17	Cyt_Nu: 14	Sec: 7	Cyt: 9	Cysk: 9	Mt: 7	Cyt	Cyt: 3	-
		19233	Chl: 13	-	Sec: 14	E.R.: 6	-	Sec: 17	-	-	Mt: 5	Sec: 4	-
		**10931**	-	-	PM: 15	Mt: 7	-	Cyt: 8.5	Mt: 7	Sec: 7	Cyt: 5	Cyt: 3	-
		27480	-	-	Cyt_Nu: 15.5	Nu: 13.5	Cyt: 12.5	Cyt: 10.5	Cysk: 9	Cyt_Nu: 8	Mt: 5	Mt: 5	-
		18090	Cyt: 6	-	Sec: 28	-	-	Sec: 18	-	-	Cyt: 3	Cyt: 2	-
		46995	Chl: 9	-	Cyt: 18	Cyt_Nu: 14.5	Nu: 7	Mt: 15.5	Cyt_Mt: 10.3	-	Cyt: 4	Cyt: 5	-
		1438	Chl: 6	-	Sec: 13	E.R.: 11	Mt: 6	Sec: 9	Mt: 7	-	Mt: 5	Sec: 4	-
α-1,3-Man transferase	2.4.1.258	11655	PM: 6	-	PM: 19	-	-	Mt: 13	PM: 9	-	Sec: 4	Sec: 1	Cyt: 1
α-1,2-Man transferase	2.4.1.259/261	**44449**	Chl: 12	-	Sec: 27	-	-	Mt: 10	PM: 10	-	Sec: 5	Sec: 1	Cyt: 1
		15548	PM: 12	-	PM: 15.5	Sec_PM: 10.5	-	PM: 17	-	-	Mt: 2	Sec: 5	-
α-1,6-Man transferase	2.4.1.260	**9095**	PM: 9	-	PM: 13	Sec: 11	-	PM: 19	-	-	Sec: 4	Sec: 1	Cyt: 1
Dolichol phosphate mannose synthase	2.4.1.83	21418	-	-	PM: 11	Cyt: 6	Mt: 6	Cyt: 8	Mt: 7	PM: 6	Cyt: 1	Cyt: 1	-
Dolichyl-phosphate beta-glucosyltransferase	2.4.1.117	15275	PM: 7	-	Sec: 20	PM: 9	-	PM: 12	Sec: 12	-	Sec: 5	Sec: 2	Cyt: 4
α-1,3-Glc transferase	2.4.1.267/265	22866	-	-	Sec: 11	PM: 9	Mt: 7	PM: 21	-	-	Cyt: 5	Sec: 5	-
		24290	PM: 7	-	Sec: 16	PM: 11	-	PM: 18	-	-	Mt: 3	Sec: 1	-
		**22282**	PM: 6	-	Sec: 9	PM: 7	Mt: 7	PM: 17	E.R.: 7		Sec: 2	Sec: 2	Cyt: 2
α-1,2-Glc transferase	2.4.1.256	20467	-	-	Sec: 15	PM: 10	-	PM: 17	-	-	Mt: 1	Mt: 3	-
Dolichyldiphosphooligosaccharide-protein glycosyltransferase	2.4.99.18	2741	PM: 13	-	PM: 22	-	-	PM: 17	E.R.: 6	-	Mt: 5	Sec: 2	-
		10761	PM: 11	-	PM: 11	Cyt: 9		PM: 26	-	-	Cyt: 2	Cyt: 2	-
		**2733**	PM: 11	-	PM: 24	-	-	PM: 23	-	-	Cyt: 5	Cyt: 4	-

Transcript numbers in bold indicate the presence of the splice leader sequence. PSORT score is the discriminant score, with larger scores having a higher probability. Scores below 5 are not reported. TargetP score is the reliability class is rated from 1 to 5 (1 is the strongest prediction and 5 is the weakest). Chl—chloroplast (green); Cyt—cytosol (grey); Cysk—Cytoskeleton; E.R.—endoplasmic reticulum (blue); Mt—mitochondria (orange); Nu—nuclear; Per—peroxisome; PM—plasma membrane (yellow); Sec—secreted or extra cellular (blue). Strength of colour indicates score.

**Table 3:** Subcellular location prediction of *E. gracilis* chloroplast marker proteins.

Marker Enzyme Name	EC Number	EG Transcript	WoLF PSORT Plant		WoLF PSORT Animal			WoLF PSORT Fungi			TargetP Plant	TargetP Nonplant	TargetP, Signal Cut
Ribulose-biphosphate carboxylase (small subunit)	4.1.1.39	7123	Chl: 13	-	Cyt: 15.5	Cyt_Nu: 14.5	Nu: 8.5	Nu: 7.5	Cyt_Nu: 7.5	Mt: 7	Cyt: 2	Cyt: 1	-
		43244	Chl: 8	-	Sec: 30	-	-	PM: 21	-	-	Sec: 3	Sec: 2	Chl: 5
		**13866**	-	-	Mt: 12.5	Cyt_Nu: 11	Cyt: 10.5	Cyt_Nu: 9.3	Cyt_Mt: 9.3	Cyt: 9	Cyt: 4	Cyt: 3	-
		59484	Chl: 7	-	Sec: 29	-	-	Nu: 10.5	Cyt_Nu: 10	Cyt: 8.5	Sec: 3	Sec: 5	Cyt: 4
NADP- glyceraldehyde-3-phosphate dehydrogenase	1.2.1.9	4454	Chl: 11	-	Mt: 19.5	Mt_Per: 11	Cyt: 6.5	Mt: 18	Cyt: 6	-	Mt: 4	Mt: 4	-
		5575	Mt: 10.5	Chl_Mt: 7.5	Mt: 28.5	Cyt_Mt: 16	-	Mt: 22.5	Cyt_Mt: 14	-	Mt: 1	Mt: 2	-
		9413	Chl: 12	-	Mt: 22.5	Mt_Per: 12	-	Mt: 23	-	-	Mt: 5	Mt: 2	-
		**7552**	Cyt: 11	-	Cyt: 24	-	-	Cyt: 22	-	-	Cyt: 2	Cyt: 1	-
		**15045**	Mt: 8.5	Chl_Mt: 7	Mt: 24	-	-	Mt: 26.5	Cyt_Mt: 14	-	Mt: 2	Mt: 2	-
Fructose-biphosphate aldolase	4.1.2.13	**15855**	Cyt: 8	-	Nu: 12.5	Cyt_Nu: 10.5	Cyt: 7.5	Cyt: 16	Mt: 6	-	Cyt: 4	Cyt: 2	-
		25832	Mt: 8.5	Chl_Mt: 7	Mt: 16	Cyt: 14	-	Mt: 24.5	Cyt_Mt: 14	-	Mt: 2	Mt: 4	-
		**7827**	Chl: 12	-	E.R.: 16	-	-	Sec: 15	-	-	Chl: 5	Sec: 4	-
		5279	Chl: 14	-	Mt: 17	E.R._Mt: 10.5	Per: 6	Mt: 15	Cyt: 6	-	Mt: 4	Mt: 3	-
Pyruvate Pi dikinase	2.7.9.1	1635	Chl: 6	-	PM: 9	Sec: 8	-	Cyt: 11.5	Mt: 9	Cyt_Nu: 7.5	Cyt: 1	Cyt: 3	-
Photosystem II D1	-	13288	PM: 6	-	PM: 12	Lyso: 10	Sec: 6	PM: 21	-	-	Sec: 1	Sec: 1	Cyt: 4
	-	10715	PM: 13	-	PM: 26.5	Sec_PM: 14	-	PM: 27	-	-	Cyt: 2	Cyt: 4	-
Photosystem II D2	-	13288	PM: 6	-	PM: 12	Lyso: 10	Sec: 6	PM: 21	-	-	Sec: 1	Sec: 1	Cyt: 4
	-	10715	PM: 13	-	PM: 26.5	Sec_PM: 14	-	PM: 27	-	-	Cyt: 2	Cyt: 4	-
Photosystem II CP47	-	899	PM: 9.5	-	PM: 31	-	-	PM: 24	-	-	Mt: 5	Sec: 5	Cyt: 2
Photosystem II CP43	-	13640	PM: 10	-	PM: 20	-	-	PM: 22	-	-	Sec: 4	Sec: 1	Cyt: 4
Cytochrome b6f	-	5228	PM: 6.5	-	PM: 12	Mt: 6	-	PM: 13	E.R.: 7	-	Cyt: 4	Cyt: 4	-
	-	23844	Chl: 13	-	Mt: 16.5	E.R._Mt: 13	Mt_Per: 12.5	Mt: 14	-	-	Cyt: 5	Mt: 5	-
Photosystem I A1	-	**158**	PM: 10	-	PM: 23	-	-	PM: 25	-	-	Mt: 5	Mt: 4	-

Transcript numbers in bold indicate the presence of the splice leader sequence. PSORT score is the discriminant score, with larger scores having a higher probability. Scores below 5 are not reported. TargetP score is the reliability class is rated from 1 to 5 (1 is the strongest prediction and 5 is the weakest). Chl—chloroplast (green); Cyt—cytosol (grey); E.R.—endoplasmic reticulum (blue); Lyso—lysosome; Mt—mitochondria (orange); Nu—nuclear; Per—peroxisome; PM—plasma membrane (yellow); Sec—secreted or extra cellular (blue). Strength of colour indicates score.

## Supplementary Materials

The following are available online at https://www.mdpi.com/2218-1989/9/6/115/s1, Table S1: Subcellular location prediction of *E. gracilis* metabolic pathway components using WoLF PSORT and TargetP1.1.
