# Supplementary material for: Euglena Central Metabolic Pathways and Their Subcellular Locations
Source: Metabolites. 2019 Jun 14;9(6):115. doi: 10.3390/metabo9060115 (PMC6630311; doi:10.3390/metabo9060115)
Supplement: Supplementary file 1 [file metabolites-09-00115-s001.pdf]

Table S1. Subcellular location prediction of *E. gracilis* metabolic pathway components using WoLF PSORT and TargetP1.1

Pathway	Enzyme name	EC number	EG transcript	WoLF PSORT Plant		WoLF PSORT Animal				WoLF PSORT Fungi			TargetP plant	TargetP nonplant	TargetP, signal cut
Glycolysis	Hexokinase	2.7.1.1	26449	Chl: 10		Sec: 13	Mt: 9			Mt: 17			Mt: 2	Mt: 2	
			31789			Cyt: 20.5	Cyt_Nu: 13			Cyt: 11.5	Mt: 9	Cyt_Nu: 7.5	Cyt: 3	Cyt: 5	
	Glucokinase	2.7.1.2	**8623**			Mt: 9.5	PM: 9	Mt_Per: 9		Cyt: 14	Mt: 8		Cyt: 3	Cyt: 2	
			22400	Chl: 10		Cyt: 11.5	Cyt_Nu: 10	Mt: 9		Cyt: 14.5	Cyt_Nu: 10		Cyt: 5	Cyt: 2	
	Fructokinase	2.7.1.4	15309	Cyt: 10		PM: 23				Cyt: 18.5	Cyt_Nu: 10		Cyt: 4	Cyt: 3	
			**19345**	Cyt: 8		PM: 12	Sec: 8			Cyt: 17			Sec: 2	Sec: 4	Cyt: 4
			13564	Chl: 11		Mt: 20				Mt: 13	Cyt: 7	Sec: 7	Mt: 1	Mt: 2	
			**14390**	Cyt: 10		Sec: 20	PM: 5			Cyt: 24			Cyt: 5	Cyt: 4	
	Phosphoglucoisomerase	5.3.1.9	**5108**	Per: 13		Cyt: 15	Cyt_Mt: 10	Nu: 7		Per: 19			Cyt: 2	Cyt: 1	
			17737	Mt: 9.5	Chl_Mt: 6.5	Cyt: 16	Cyt_Nu: 11.5	Mt: 8		Cyt: 8.5	Mt: 8	Cyt_Nu: 7	Cyt: 5	Cyt: 4	
			**8453**	Cyt: 9		Cyt: 17				Cyt: 13	Per: 6		Cyt: 2	Cyt: 2	
			19460	Cyt: 9.5		Cyt: 11.5	Cyt_Nu: 10.5	Mt: 9		Sec: 20			Sec: 5	Cyt: 4	
	Phosphofructokinase	2.7.1.11	**6957**			Cyt: 15.5	Cyt_Nu: 13	Nu: 9.5		Cyt: 16	Cyt_Nu: 13	Nu: 6	Cyt: 2	Cyt: 1	
			6970			Cyt: 14	Nu: 11			Cyt: 17	Cyt_Nu: 11		Cyt: 1	Cyt: 2	
			12862			Nu: 10	Cyt_Nu: 9.5	PM: 7	Cyt: 7	Cyt: 8	Mt: 6	PM: 6	Cyt: 5	Cyt: 4	

			**7619**	Cyt: 9		Cyt: 12	Nu: 8	PM: 6	Cyt: 17	Cyt_Nu: 12.5	Nu: 6	Cyt: 2	Cyt: 1	
			**7325**	Cyt: 8		Cyt: 15.5	Cyt_PM: 9	Mt_Per: 6	Cyt: 13	Cyt_Nu: 8	Mt: 7	Cyt: 3	Cyt: 2	
			1546	Cyt: 6		Cyt: 15	Nu: 8		Cyt: 12	Mt: 10		Cyt: 3	Cyt: 4	
	Diphosphate-fructose-6-phosphate 1-phosphotransferase	2.7.1.90	**7325**	Cyt: 8		Cyt: 15.5	Cyt_PM: 9	Mt_Per: 6				Cyt: 2	Cyt: 3	
			12862			Nu: 10<br>Cyt_Nu: 9.5	PM: 7	Cyt: 7<br>Mt: 6	Cyt: 13	Cyt_Nu: 8	Mt: 7	Cyt: 4	Cyt: 5	
			6970			Cyt: 14	Nu: 11		Cyt: 8	Mt: 6	PM: 6	Cyt: 2	Cyt: 1	
	Fructose-bisphosphate aldolase	4.1.2.13	**15855**	Cyt: 8		Nu: 12.5	Cyt_Nu: 10.5	Cyt: 7.5	Cyt: 16	Mt: 6		Cyt: 4	Cyt: 2	
			7827	Chl: 12		E.R.: 16			Sec: 15			Chl: 5	Sec: 4	
			5279	Chl: 14		Mt: 17	E.R._Mt: 10.5	Per: 6	Mt: 15	Cyt: 6		Mt: 4	Mt: 3	
			25832	Mt: 8.5	Chl_Mt: 7.5	Mt: 16	Cyt: 14		Mt: 24.5	Cyt_Mt: 14		Mt: 2	Mt: 4	
	Triosephosphate isomerase	5.3.1.1	**14120**	Chl: 13		Mt: 21	Cyt: 8		Mt: 18			Mt: 5	Mt: 2	
			**18179**	Cyt: 10		Cyt: 24.5	Cyt_Nu: 14		Cyt: 20	cysk: 6		Cyt: 2	Cyt: 2	
			30563	Cyt: 7		Cyt: 15.5	Cyt_Nu: 15.5	Nu: 6.5	Mt: 16	Cyt: 7.5	Cyt_Nu: 5.5	Cyt: 1	Cyt: 1	
			**44253**			Cyt: 11	Cyt_Nu: 11	Sec: 8<br>Mt: 8	Mt: 22			Cyt: 3	Cyt: 3	
			30456	Chl: 14		Mt: 24			Mt: 15	Sec: 9		Chl: 3	Mt: 5	

	Glyceraldehyde 3-phosphate dehydrogenase	1.2.1.12	16406	Cyt: 13		Cyt: 28				Cyt: 27			Cyt: 5	Sec: 5	
			**12332**	Per: 9		Cyt: 18.5	Cyt_Nu: 14	Nu: 8.5		Per: 16	Cyt: 9	Cyt_Nu: 6	Cyt: 1	Cyt: 2	
			**8042**			E.R.: 16.5	E.R._ Mt: 11	Sec: 9		Per: 13	Cyt: 7		Sec: 4	Sec: 4	Chl 5
	Phosphoglycerate kinase/ phosphoglycerate mutase	2.7.2.3/ 5.4.2.1	**10778**	Cyt: 8		Cyt: 21.5	Cyt_Nu: 13			Cyt: 18	Mt: 8		Cyt: 1	Cyt: 2	
			12260	Cyt: 9		Cyt: 20	Cyt_Nu: 16.3	Cyt_Per: 11.6	Nu: 7.5	Cyt: 20.5	Cyt_Nu: 12.5		Cyt: 2	Cyt: 1	
			12119	Cyt: 10		Cyt: 21	Cyt_Nu: 17	Nu: 9		Cyt: 25			Cyt: 3	Cyt: 1	
			32014	Chl: 11		Mt: 18	Sec: 10			Mt: 13	Cyt: 6		Sec: 5	Mt	Cyt: 5
	Enolase	4.2.1.11	**7256**	Chl: 14		Sec: 9				Sec: 12	Mt: 8	Cyt: 6	Mt: 4	Sec: 3	
			**12376**	Cyt: 6		Cyt: 25.5	Cyt_Nu: 15.5			Cyt: 24.5	Cyt_Nu: 13.833	Cyt_Per: 13.666	Cyt: 4	Cyt:1	
			6665	Cyt: 7		Cyt: 20.5	Cyt_Nu: 12			Cyt: 20.5	Cyt_Nu: 12		Cyt:3	Cyt: 2	
	Pyruvate kinase	2.7.1.40	7941	Cyt: 9		Cyt: 15.5	Cyt_Nu: 11	Nu: 5.5		Cyt: 14	Mt: 10		Cyt: 3	Cyt: 3	
			6211	Chl: 9		Mt: 15	Sec: 6			Mt: 16	Cyt: 7		Mt: 2	Mt: 4	
			2957			Cyt: 23	Cyt_Nu: 16.8	Cyt_plas: 13.1667		Cyt: 15.5	Cyt_Nu: 11.5	Nu: 6.5	Cyt: 2	Cyt: 2	
			6124	Chl: 5		Cyt: 12.5	Cyt_Nu: 10	Nu: 6.5		Cyt: 18.5	Cyt_Nu: 10	Mt: 8	Cyt: 4	Cyt: 4	
	Pyruvate dehydrogenase ($NADP^+$)	1.2.1.51	**9827**	Cyt: 9		Mt: 14	Cyt: 11.5	Cyt_Nu: 11.5	Nu: 6.5	Mt: 15.5	Cyt_Mt: 13	Cyt: 9.5	Mt: 2	Mt: 4	

			**8975**	Mt: 11.5	Chl_Mt: 7	Mt: 22.5	Cyt_Mt: 15	Cyt: 6.5		Mt: 26				Mt: 2	Mt: 4	
			**14931**	Per: 7		Mt: 14	Cyt: 11.5	Cyt_Nu: 8.5		Per: 14	Cyt: 9	Cyt_Nu: 6		Mt: 5	Cyt: 5	
			**14495**	Mt: 9		Mt: 21	Cyt: 8			Mt: 27				Mt: 1	Mt: 2	
	Dihydrolipoyl transacetylase	2.3.1.12	**56241**	Chl: 10		Mt: 21				Mt: 20.5	Cyt_Mt: 14	Cyt: 6.5		Mt: 3	Mt: 1	
	Dihydrolipoyl dehydrogenase	1.8.1.4	**6933**			Mt: 27.5	Cyt_Mt:15			Mt: 20				Mt: 3	Mt: 1	
Gluconeogenesis	Phosphoenolpyruvate synthase	2.7.9.2	**1653**	Cyt: 8		Cyt: 16.5	Cyt_Nu: 14	Cyt_Mt: 10.2	Cyt_PM: 9.5	Cyt: 15.5	Cyt_Nu: 10.5			Cyt: 3	Cyt: 1	
	Phosphoenolpyruvate carboxykinase	4.1.1.32	535	Chl: 6		Cyt_Nu: 15.5	Nu: 15	Cyt: 12		Cyt: 13.5	Cyt_Per: 9.5			Cyt: 4	Cyt: 2	
			4483	Chl: 14		Mt: 19	Cyt_Mt: 11			Mt: 18				Mt: 4	Sec: 5	
			14414	Cyt: 6.5		Cyt: 22	Nu: 6			Cyt: 17.5	Cyt_Nu: 12.8	Cyt_Per: 10.7	Nu: 6	Cyt: 2	Cyt: 2	
	Fructose-1,6-bisphosphatase	3.1.3.11	8727	PM: 9		Sec: 10	Mt: 7	lyso: 7		PM: 8	Sec: 7	Mt: 6		Sec: 5	Sec: 3	Chl: 5
			**15632**	Cyt: 7		Cyt_Nu: 11.5	Cyt: 10	Nu: 7	Sec: 6	cysk: 14	Cyt: 6.5	Cyt_Nu: 6		Cyt: 4	Cyt: 2	
			**7683**	Chl: 13		Nu: 7				Sec: 19				Chl: 5	Cyt: 3	
			**13690**	Chl: 5		Sec: 7	E.R.: 6			Cyt: 16	Cyt_Nu: 10.5			Chl: 5	Cyt: 3	
			**4570**	Chl: 11		PM: 17				Sec: 10	Mt: 7			Cyt: 5	Sec: 5	
TCA cycle	Citrate synthase	4.1.3.7	10302	Per: 11		Cysk: 17	Cyt: 11			Per: 17	Cyt: 6			Cyt: 2	Cyt: 1	

			6849	Per: 12		Cyt: 15	Cyt_Nu: 12.5	Nu: 8	Per: 17	Cyt: 6		Cyt: 2	Cyt 1:	
			11595	Per: 7		Cyt: 13	Nu: 9		Per: 15			Cyt: 5	Cyt: 2	
			**2898**	Chl: 7	Mt: 6	Mt: 23.5	Mt_Per: 13		Mt: 23.5	Cyt_Mt: 13		Mt: 2	Mt: 2	
	Aconitase	4.2.1.3	**2608**	Cyt: 10		Cyt: 16.5	Cyt_Nu: 11		Cyt: 24			Cyt: 2	Cyt: 3	
			**1951**	Mt: 10		Nu: 15.5	Mt: 12	Cyt_Nu: 10	Mt: 24			Mt: 4	Mt: 1	
	Isocitrate dehydrogenase	1.1.1.41, 1.1.1.42	6875	Cyt: 7		Cyt: 17	Mt: 15		Cyt: 22.5	Cyt_Nu: 12		Cyt: 3	Cyt: 2	
			**10115**	Chl_Mt: 7.5	Mt: 7	Mt: 29	Cyt_Mt: 16.5		Mt: 22			Mt: 1	Mt: 1	
			10872	Mt: 10		Mt: 28			Mt: 26.5	Cyt_Mt: 14		Mt: 3	Mt: 4	
			**10979**			Mt: 24	Cyt_Mt: 15.3	Mt_Per: 12.8<br>Cyt: 5.5	Per: 15	Cyt: 7.5		Mt: 2	Mt: 2	
	2-Oxoglutarate decarboxylase	4.1.1.71	**121**	Nu: 8	PM: 7	Nu: 8	PM: 7	Cyt: 7	Cyt: 12.5	Cyt_Nu: 7.5	Mt: 6	Cyt: 3	Cyt: 3	
	Succinic semialdehyde dehydrogenase	1.2.1.16	7552	Cyt: 24		Cyt: 24			Cyt: 22			Cyt: 2	Cyt: 1	
			5575	Mt: 28.5	Cyt_Mt: 16	Mt: 28.5	Cyt_Mt: 16		Mt: 22.5	Cyt_Mt: 14		Mt: 1	Mt: 2	
			**3780**	Mt: 10.5	Cyt_Mt: 10.3	Mt: 10.5	Cyt_Mt: 10.3	Cyt: 9	Mt: 16	Cyt: 7.5	Cyt_Nu: 5.5	Mt: 5	Mt: 4	
			**5750**	Mt: 27.5	Cyt_Mt: 16.5	Mt: 27.5	Cyt_Mt: 16.5		Mt: 25.5	Cyt_Mt: 14		Mt: 3	Mt: 2	
	Succinate dehydrogenase	1.3.5.1	7873	PM: 6		PM: 31			PM: 23			Cyt: 1	Cyt: 4	

	Fumarase	4.2.1.2	**4764**	Chl: 8		Mt: 27.5	Cyt_Mt: 17			Mt: 26			Mt: 1	Mt: 2	
	Malate dehydrogenase	1.1.1.37	12212	Cyt: 11		Cyt: 21	Cyt_Nu: 13.5	Mt: 7		Cyt: 15			Cyt: 4	Cyt: 3	
			15919	Cyt: 10		Mt: 16	Cyt: 10.5	Cyt_Nu: 6		Cyt: 10	Sec: 8	Mt: 7	Cyt: 5	Cyt: 5	
Dicarboxylic acid bypass	Malate dehydrogenase (NADP-specific oxaloacetate-decarboxylating)	1.1.1.40	8836	Nu: 6	Mt: 5	Mt: 17	Cyt: 8			Mt: 21			Mt: 2	Sec: 5	
			7652	Cyt: 9		PM: 9	Sec: 6	Cyt_Nu: 5.5		Cyt: 8.5	Cyt_Nu: 6.5	Cysk: 6	Cyt: 5	Cyt: 4	
	NAD-specific malate dehydrogenase	1.1.1.39	5925	Cyt: 8		Cyt_Nu: 12.5	Cyt: 12	Nu: 7	Mt: 10	Cyt: 6.5	Cyt_Nu: 5.5		Cyt: 3	Cyt: 1	
			7652	Cyt: 9		PM: 9	Sec: 6	Cyt_Nu: 5.5		Cyt: 8.5	Cyt_Nu: 6.5	Cysk: 6	Cyt: 5	Cyt: 4	
	Phosphoenolpyruvate carboxylase	4.1.1.31	1597	Nu: 6		Cyt_Nu: 10.5	Nu: 10	Cyt: 9		Nu: 8	Cyt: 7	Cysk: 6	Cyt: 2	Sec: 5	
	Pyruvate carboxylase	6.4.1.1	12535	Cyt: 9		Mt: 20				Mt: 15	Cyt: 6.5	Cyt_Nu: 6	Cyt: 3	Cyt: 2	
			23270	Cyt: 8		Cyt: 19.5	Cyt_Nu: 12.5	Mt: 8		Cyt: 14.5	Mt: 11	Cyt_Nu: 8.5	Cyt: 5	Cyt: 3	
			724	Nu: 6		Cyt: 20.5	Cyt_Nu: 12			Cysk: 17	Cyt: 8.5	Cyt_Nu: 5.5	Cyt: 3	Cyt: 5	
			36271	Cyt: 9		Cyt_Nu: 14.5	Nu: 12.5	Cyt: 11.5	Cyt_Nu: 11	Cyt: 10	Nu: 8		Cyt: 2	Cyt: 5	
			**97**	Cyt: 8		Mt: 21				Mt: 26			Mt: 5	Mt: 2	

C2 metabolism	Alcohol dehydrogenase	1.1.1.1	13473	Cyt: 11		Sec: 14	Cyt: 11.5	Cyt_Nu: 9.5		Cyt: 27			Cyt: 1	Cyt: 2	
			21676	Per: 12		Cyt: 13.5	Sec: 11	Cyt_Nu: 10.5		Per: 18	Cyt: 7		Cyt: 1	Cyt: 2	
	Acetaldehyde dehydrogenase	1.2.1.10	**8156**	Chl: 7		Mt: 18.5	Cyt_Mt: 11	PM: 6		Mt: 14	Sec: 6		Mt: 1	Mt: 2	
	Acetyl-CoA synthetase	6.2.1.1	**3254**	PM: 8		PM: 16	E.R.: 7			PM: 14			Cyt: 3	Cyt: 4	
			**4603**	Cyt: 7	E.R.: 5	Cyt: 18.5	Cyt_Nu: 12.5	Nu: 5.5		Cyt: 18			Cyt: 3	Cyt: 2	
			9669	Cyt: 9		Cyt: 16	Cyt_Nu: 13	Nu: 8		PM: 16	Cyt: 7		Cyt: 2	Cyt: 1	
			**3515**	Pero: 10		PM: 10	Mt: 8	Per: 5.5	Cyt_Per: 5.2	Per: 16	Cyt: 7.5		Sec: 2	Cyt: 5	Cyt: 4
Glyoxylate	Isocitrate lyase	4.1.3.1	**715**	Chl: 7	Cyt: 6	Mt: 16.5	Mt_Per: 10	Nu: 7.5	Cyt_Nu: 6.5	Mt: 19.5	Cyt_Mt: 12.5		Mt: 1	Mt: 2	
	Malate synthase	2.3.3.9	729	Cyt: 11		Cyt_Nu: 14.2	Nu: 13	Cyt: 11	Cyt_PM: 7	Cyt: 14.5	Cyt_Nu: 10.5	Nu: 5.5	Cyt: 3	Cyt: 2	
			**2530**	Cyt: 12		Cyt: 16.5	Cyt_Nu: 10	Sec: 7		Cyt: 22	Cyt_Nu: 13		Cyt: 2	Cyt: 2	
Oxidative PPP	Glucose-6-phosphate 1-dehydrogenase	1.1.1.49	10052	Cyt: 7		Cyt_Nu: 17	Nu: 16	Cyt: 16		Nu: 8.5	Cyt_Nu: 8	Per: 7	Cyt: 2	Cyt: 2	
	6-Phosphogluconolactonase	3.1.1.31	**4334**	Mt: 9.5	Cyt_Mt: 5.5	Mt: 17	Cyt_Nu: 8.5	Cyt: 7.5		Mt: 14	Cyt: 7	Cyt_Nu: 6.833	Cyt: 5	Cyt: 5	
	6-Phosphogluconate dehydrogenase	1.1.1.44	**7631**			PM: 15				Cyt: 12.5	Cyt_Nu: 7.5		Sec: 3	Cyt: 4	

Nonoxidative PPP	Pentose-5-phosphate-3-epimerase	5.1.3.1	**19182**			Cyt: 10.5	Cyt_Nu: 9.5	Mt: 9		Sec: 12	Cyt: 6		Cyt: 4	Cyt: 3	
			23949	Chl: 5.5	Chl_Mt: 5.5	Mt: 18	Cyt: 12.5	Cyt_Nu: 7		Cyt: 18	Mt: 8		Cyt: 5	Mt: 4	
	Ribose 5-phosphate isomerase	5.3.1.6	**15619**			PM: 14.5	Cyt: 9.5	Sec_PM: 8	Cyt_Nu: 6.5	Cyt: 16	Cyt_Nu: 10.5		Cyt: 4	Cyt: 4	
			**20009**	Cyt: 12		Nu: 14.5	Cyt_Nu: 14.5	Cyt: 11.5		Mt: 14	Cyt: 10.5	Cyt_Nu: 6.5	Cyt: 5	Cyt: 5	
	Transketolase	2.2.1.1	**2997**	PM: 8.5	Cyt_plas: 5.5	E.R.: 19				PM: 7	Cyt: 5.5		Sec: 4	Cyt: 4	Cyt:5
			3894	Chl: 7		PM: 8	Cyt: 7	Nu: 6		Cyt: 12.5	Mt: 11	Cyt_Nu: 7	Cyt: 4	Cyt: 4	
			**4605**	Cyt: 7		Cyt: 11.5	Cyt_Nu: 9	Sec: 6	Nu: 5.5	Cysk: 12	Cyt: 11.5	Cyt_Nu: 7	Cyt: 4	Cyt: 4	
	Transaldolase	2.2.1.2	13075	Cyt: 6		Nu: 16	Cyt: 11			Cyt: 19			Cyt: 3	Cyt: 2	
			**8390**	Chl: 8		Cyt: 18.5	Cyt_Nu: 11.5			Mt: 9	Cyt: 8.5	Cyt_Nu: 6.5	Cyt: 4	Cyt: 5	
Glycolate	Glycolate dehydrogenase	1.1.99.14	**8383**	Per: 8		Mt: 25				Per: 15	Cyt: 8	Cyt_Nu: 5.5	Mt: 2	Mt: 3	
			**11827**			Mt: 22				Mt: 16			Mt: 2	Mt: 2	
Paramylon synthesis	1,3-β-Glucan synthase	2.4.1.34	73	PM: 13		PM: 21	E.R.: 6			PM: 20			Sec: 5	Sec: 1	Cyt: 5
			11758	PM: 8		PM: 15	Cyt: 5.5			PM: 19			Cyt: 2	Cyt: 1	
	Endo-1,3-β-glucanases	3.2.1.6	183	PM: 10.5	Golg_PM: 6.5	PM: 22	E.R.: 10			PM: 24			Sec: 5	Sec: 2	Chl: 2

			2352	Chl: 14		Nu: 15.5	Cyt_Nu: 12	Cyt: 7.5	PM: 5.5	Mt: 14	Cyt: 7.5	Cyt_Nu: 5.5	Chl: 2	Cyt: 4	
	Glucan endo-1,3-β-glucanases	3.2.1.39	11364	PM: 6		Sec: 16	PM: 7	E.R.: 6		Sec: 12	PM: 10		Sec: 4	Sec: 1	Cyt: 4
			11498	Chl: 8		Nu: 19	Cyt: 6			Cyt: 15.5	Cyt_Nu: 10.5		Cyt: 3	Cyt: 2	
			3590	Chl: 11		Mt: 16.5	E.R. Mt: 9	Cyt_Nu: 5.7	Cyt_Per: 5.2	Cyt: 12	Sec: 12		Cyt: 4	Cyt: 2	
			6201	E.R.: 5		Cyt: 13	PM: 9			Mt: 10	Cyt: 9.5	Cyt_Nu: 6.5	Cyt: 2	Cyt: 2	
	Exo-1,3-β-glucanases	3.2.1.58	11628			Cyt: 12	Mt: 8.5	E.R.: 6	Mt_Per: 5.5	Mt: 6	Cyt: 6		Cyt: 2	Sec: 5	
			7060	E.R.: 6		PM: 25.5	Sec_PM: 13.5			PM: 15	Mt: 5		Cyt: 1	Cyt: 3	
Trehalose synthesis	Trehalose-phosphate synthase/ trehalose-phosphate phosphatase	2.4.1.15/ 3.1.3.12	**2409**	Cyt: 8		Cyt: 19.5	Cyt_Nu: 13	Sec: 6	Nu: 5.5	Cysk: 11	Cyt: 6		Chl: 5	Mt: 3	
	Trehalose synthase	2.4.1.245	8134	PM: 7		PM: 20				PM: 16			Cyt: 2	Cyt: 2	
			10436			PM: 23				Cyt: 13.5	Cyt_Nu: 11.5	Nu: 6.5	Cyt: 2	Cyt: 2	
			3686	Chl: 13		Mt: 24				Mt: 6	PM: 6	Sec: 6	Mt: 4	Cyt: 5	
Methionine synthesis	Cobalamin-dependent methionine synthase	2.1.1.13	3052	Cyt: 9		Cyt: 16.5	Cyt_Nu: 12	Nu: 6.5		Cyt_Mt: 13.5	Mt: 13	Cyt: 12	Cyt: 3	Cyt: 2	

			904			PM: 13	Cyt: 8		Cyt: 15.5	Cyt_Nu: 9.5		Cyt: 2	Cyt: 2	
			1784	Cyt: 8		Cyt: 17	Nu: 7		Cysk: 20	Cyt: 6		Cyt: 1	Cyt: 1	

Transcript numbers in bold indicate the presence of the splice leader sequence. PSORT score is the discriminant score, with larger scores having a higher probability. Scores below 5 are not reported. TargetP score is the reliability class is rated from 1 to 5 (1 is the strongest prediction and 5 is the weakest). Chl – Chloroplast (green); Cyt – Cytosol (grey); Cysk – Cytoskeleton; E. R. – Endoplasmic Reticulum (Blue); Lyso – Lysosome; Mt – Mitochondria (Orange); Nu – Nuclear; Per – Peroxisome; PM – Plasma Membrane (Yellow); Sec – Secreted or extra cellular (Blue). Strength of colour indicates score.
